# Correlation between audiometric data and the 35delG mutation in ten patients

**DOI:** 10.1016/S1808-8694(15)31174-5

**Published:** 2015-10-19

**Authors:** Vânia Belintani Piatto, Otávio Augusto Vasques Moreira, Magali Aparecida Orate Menezes da Silva, José Victor Maniglia, Márcio Coimbra Pereira, Edi Lúcia Sartorato

**Affiliations:** 1 PhD, Adjunct Professor - Department of Otorhinolaryngology / Head and Neck Surgery - FAMERP; 2 5th Year Medical Student - FAMERP; 3 M.Sc. Head of the Speech and Hearing Therapy Division - Department of Otorhinolaryngology / Head and Neck Surgery - FAMERP; 4 Associate Professor - Head of the Department of Otorhinolaryngology / Head and Neck Surgery - FAMERP; 5 M.Sc. Department of Otorhinolaryngology / Head and Neck Surgery - FAMERP; 6 PhD. Head of the Center for Molecular Biology and Genetic Engineering - CBMEG-UNICAMP; Faculdade de Medicina de São José do Rio Preto, SP - FAMERP

**Keywords:** molecular analysis, audiometry, hearing loss, 35delg mutation

## Abstract

Mutations in the connexin 26 gene seem to be extremely common in non-syndromic hereditary deafness genesis, especially the 35delG, but there are still only a few studies that describe the audiometric characteristics of patients with these mutations.

**Aim:**

to analyze the audiometric characteristics of patients with mutations in the connexin 26 gene in order to outline genotype-phenotype correlation.

**Materials and Methods:**

Tonal audiometries of 33 index cases of non-syndromic sensorineural hearing loss were evaluated and eight affected relatives. Specific molecular tests were carried out to analyze mutations in the connexin 26 gene. **Experiment Design**: Retrospective, cross-sectional study.

**Results:**

A 27.3% prevalence of mutation 35delG was found in the index cases and 12.5% among the relatives affected. In relation to hearing loss degree, 41.5% of the patients were found with profound hearing loss, 39% with severe HL and 19.5% with moderate HL with homozygote and heterozygote patients for the 35delG predominating in the severe-moderate hearing losses.

**Conclusion:**

Our results suggest that the audiometric data associated with the molecular diagnose of hearing loss helped us to outline a genotype-phenotype correlation in ten patients with 35delG mutation. However, it is still necessary to run multicentric studies to verify the real phenotypic expression in the Brazilian population, as far as the 35delG mutation is concerned.

## INTRODUCTION

It is well known that in developed countries one in 750 births is likely to produce a child with sensorineural hearing impairment and it is estimated that one in 1000 children is affected by severe deafness at birth or by the end of the prelingual period^1^. Approximately 60% of all causes of prelingual deafness can be attributed to genetic factors. Therefore, the genetic etiology is becoming increasingly relevant in cases of hearing impairment and/or deafness. The remaining 40% are distributed over a vast variety of etiologies^2^. There are three identifiable kinds of inheritance pattern for the inherited deafness: autosomal recessive, autosomal dominant, and X-linked. From 75% to 85% of cases of non-syndromic prelingual deafness are manifested as autosomal recessive forms. Autosomal dominant forms account for about 15% to 25% of cases, and the remaining 1% to 3% are of X-linked Mendelian inheritance. There are also descriptions of forms which are inherited exclusively from the mother, corresponding to mitochondrial inheritance, associated or not with autosomal dominant inheritance^2^,^3^.

In terms of phenotype, the autosomal recessive forms are more severe, being responsible for almost all forms of congenital deafness. These, in turn, are caused in great part by cochlear defects, leading to sensorineural hearing impairment. The autosomal dominant forms seem to contribute more to cases of postlingual deafness, which are usually progressive, and the impairment is, in most cases, conductive or mixed (conductive and sensorineural)^4^,^5^.

Mutations in the conexin 26 or GJB2 - Gap Junction Protein Beta 2 gene, located on the long arm of chromosome 13 (13q11-12), appear to be extremely common in the genesis of inherited non-syndromic deafness, and account for 34% to 50% of autosomal recessive sensorineural deafness (DFNB1) and for 10% to 37% of sporadic cases^6^, ^7^, ^8^, ^9^, ^10^.

Deletion of a guanine out of a sequence of six guanines going from position 30 to position 35 in the conexin 26 (GJB2) gene is the mutation that occurs in 60% to 80% of cases. This nucleotide deletion can occur at position 35 (35delG) or at position 30 (30delG) of the gene, but the deletion at position 35 (35delG), genetically related to chromosome 13, is the more frequent one, varying between approximately 50% to 85% of the non-syndromic deafness cases in patients from Italy, Spain, and Israel^7^,^11^, ^12^, ^13^. n a study with Brazilian patients14, mutations in gene GJB2 were found in 22% of families tested with at least one patient with hearing loss, and in 11.5% of cases in which an environmental etiology was not completely ruled out. In the cases in which this mutation in conexin 26 gene occurs, the gene product, a protein named conexin, no longer exerts its functions correctly, while the cochlea is structurally normal^15^.

Even though the GJB2 gene is of great importance in the etiology of non-syndromic sensorineural impairment, there are so far very few studies describing the audiometric characteristics of patients with mutations in the GJB2 gene associated with hearing loss, especially in Brazil. According to these studies, in patients who are homozygous for mutations in the GJB2 gene and mainly in those who are homozygous for 35delG, the hearing loss is characterized for being prelingual, affecting all frequencies, being non-progressive, varying in degree from moderate to deep, even among impaired siblings of the same family, and not being associated with vestibular alterations and radiological abnormalities of the inner ear^16^,^17^. The present study had the objective of analyzing the audiometric characteristics of patients with mutations in the conexin 26 gene, in order to outline a genotype-phenotype correlation.

## PATIENTS AND METHOD

During the period from March through June, 2000, a cross-section study was carried out, in which 33 index cases (23 male and 10 female) with non-syndromic sensorineural hearing impairment were investigated. They aged between 3 and 37 years, were randomly selected at the Ear-Nose-Throat Outpatient Clinic. Of these index cases, eight relatives were evaluated (4 male and 4 female), aged 11 to 45 years, who also presented non-syndromic sensorineural hearing impairment. Hence, we evaluated 33 families with at least one member with hearing loss, totaling 41 impaired individuals. This study was approved by the Research Ethics Committee, Protocol No. 4429/2000. The complete history of each patient was obtained to investigate age at onset of the hearing impairment, presence of other cases in the family, and to exclude the possibility of environmental causes such as, maternal-fetal infections, perinatal complications, meningitis, use of ototoxic drugs, acoustic trauma. Physical, otorhinolaryngological and systemic examinations, as well as complementary tests, were carried out to exclude signs suggesting syndromic forms of hearing loss (especially craniofacial dysmorphism, skin disorders, anomalies of branchial, cardiac or thyroidal origin, vision disorders, etc.). Moreover, patients were submitted to ophthalmologic evaluation (including fundoscopy), vestibular tests and computerized tomography of the temporal bone. Thus, a complete clinical evaluation was performed to exclude patients with hearing loss caused by environmental factors, congenital malformations of the inner ear or genetic syndromes. The patients were audiologically tested by pure-tone audiometry, performed at the Speech Therapy Outpatient Clinic, and those with non-syndromic sensorineural hearing impairment classified as mild (25-40 dB), moderate (41-60 dB), severe (61-80 dB) or profound (>81 dB) were included^18^.

Molecular analysis was carried out at the Molecular Biology Center, after DNA extraction from whole blood, done at the Genetics and Molecular Biology Research Unit, using a genomic DNA extraction kit (GFXTM Genomic Blood DNA Purification Kit, Amersham Pharmacia Biotech Inc.), according to the manufacturer protocol. To detect the 35delG mutation, the technique used was allele-specific polymerase chain reaction (AS-PCR) with specific primers8. Primers named control A (direct) and B (reverse) were also synthesized, for co-amplification of the GJB2 gene with a segment of the amelogenin gene homologous to the X-Y chromosome, thus being used as internal amplification controls19. Additionally, the PCR technique was used to detect the Delta (GJB6 - D13S1830) mutation, with specific primers20, in heterozygous samples and in those who did not present the 35delG mutation. The AS-PCR and PCR products were analyzed by electrophoresis on 1.5% agarose gel in TBE 1X buffer, containing ethidium bromide, at a concentration of 0.5mg/mL, submitted to ultraviolet light to confirm that the reaction was successful, and the gel was photographed. The samples which did not present the mutations under study on both alleles, or heterozygous samples, were submitted to automated sequencing.

Two pairs of primers were synthesized^21^ and amplification of the GJB2 gene in two fragments was obtained by the PCR reaction prior to sequencing. The amplified fragments were purified using the Wizard SV Gel and PCR Clean-UP System Kit (PROMEGA), and the sequencing reactions were run in both directions in an ABI PRISMTM 377 automated sequencer (Perkin Elmer) using the BigDyeTM Terminator Cycle Sequencing Kit V2.0 Ready Reaction (ABI PRISM/PE Biosystems). The sequences obtained were analyzed and compared with the normal sequence, using the Gene Runner V3.05 program to align the nucleotide sequences and the Chromas V1.45 program to edit the electropherograms.

The present retrospective study was submitted to and approved by the Research Ethics Committee, Protocol No. 2813/2004, in order to review the molecular and audiometric data of patients.

Percentages with their respective standard deviations were calculated, and the results are expressed in % (SD%).

## RESULTS

Table 1 presents the overall genotype results obtained after molecular analysis performed in the 33 index cases and 8 affected relatives. The clinical and audiometric data concerning the index cases, such as gender, age, time of onset and degree of hearing loss, and familial recurrence, are shown in Chart 1.

**Table 1:** Distribution of index cases (n) and affected relatives (n) in relation to the genotypes found by molecular analysis of the GJB2 gene and the Delta (GJB6 - D13S1830) mutation.

Genotype	Index cases n (33) %		Affected relatives n (8) %	
Homozygous 35delG	5	15.2	0	0
Heterozygous 35delG	2	6.1	0	0
Composite heterozygous 35delG/V37I	1	3.0	0	0
Composite heterozygous 35delG/D(GJB6-D13S1830)	1	3.0	1	12.5
Total	9	27.3	1	12.5
No mutation	24	72.7	7	87.5
Total	33	100	8	100

**Chart 1:** Clinical and audiometric data of the 33 index cases of the study and 8 affected relatives (n=41) who were submitted to molecular analysis of the GJB2 gene and the Delta (GJB6-D13S1830) mutation.

Index cases (IC)	Sex	Age (years)	Onset of HL	Degree of HL	Familial Recurrence	Age (years)	Degree of HL
1	M	15	Prelingual	Severe			
2	F	12	Prelingual	Profund	brother CI31	11	Moderate
3	M	9	Prelingual	Severe			
4	F	28	Postlingual	Profund			
5	F	3	Prelingual	Profund			
6	M	8	Prelingual	Severe			
7	M	4	Prelingual	Profund			
8	F	10	Postlingual	Severe			
9	M	7	Prelingual	Profund			
10	M	3	Prelingual	Severe			
11	M	3	Prelingual	Severe			
12	M	9	Prelingual	Severe			
13	F	3	Prelingual	Profund			
14	M	9	Prelingual	Profund			
15	M	6	Prelingual	Severe			
16	M	5	Prelingual	Profund			
					sister CI18	28	Moderate
17	F	33	Prelingual	Severe	brotherCI19	37	Profund
					brotherCI20	19	Profund
21	M	37	Postlingual	Profund			
22	M	8	Prelingual	Severe			
23	M	15	Prelingual	Severe			
24	F	3	Prelingual	Severe			
25	M	4	Prelingual	Profund			
26	F	9	Prelingual	Moderate			
27	F	5	Prelingual	Profund			
28	M	10	Prelingual	Moderate	mãe CI29	38	Moderate
30	M	14	Postlingual	Severe			
32	M	17	Prelingual	Profund	sister CI33	26	Profund
34	F	14	Prelingual	Severe	brotherCI35	35	Severe
36	M	32	Prelingual	Profund			
37	M	7	Prelingual	Profund	mãe CI38	45	Moderate
39	M	15	Prelingual	Moderate			
40	M	6	Prelingual	Severe			
41	M	16	Prelingual	Moderate			

(IC)- Index cases; (M)- Male; (F)- Female; (HL)- Hearing loss

Therefore, we found a prevalence of 27.3% (SD%=7.7) for the 35delG mutation in the index cases analyzed (9/33), of 21.2% (SD%=5.03) of the alleles (14/66) with the 35delG mutation, and of 12.5% (SD%=11.69) in the affected relatives. For each mutation, V37I and Delta (GJB6-D13S1830), a 3% prevalence (SD%=2.96) was found.

The audiometric tests performed in patients with hearing impairment (n=41) showed the following results with regard to degree of loss: profound − 17 patients (41.5%, SD%=7.69); severe − 16 patients (39.0%, SD%=7.61) and moderate − 8 patients (19.5%, SD%=6.18). Of the five patients who were homozygous for 35delG, one presented a profound degree (2.43%, SD%=2.40), three presented a severe degree (7.31%, SD%=4.06), and one patient presented a moderate degree of hearing loss (2.43%, SD%=2.40). Of the two patients who were heterozygous for 35delG, one (2.43%, SD%=2.40) presented a severe and the other (2.43%, SD%=2.40) a moderate degree of hearing loss. The patient who was a 35delG/V37I composite heterozygote (2.43%, SD%=2.40) and the two 35delG/Delta (GJB6-D13S1830) composite heterozygotes (4.87%, SD%=3.36) presented a severe degree of hearing loss. The predominant audiometric frequencies were 4000 to 8000 Hertz (Hz). All patients with the 35delG mutation presented hearing loss with prelingual onset.

The phenotype/genotype correlation of the index cases and affected relatives, with the diagnosed mutation, is represented in Chart 2.

**Chart 2:** Phenotype and genotype of the index cases and affected relatives with mutations in the GJB2 gene and with the Delta (GJB6-D13S1830) mutation

Index cases (IC)	Onset HL	Degree	Mutation Allele 1/Allele 2	Familial Recurrence with Mutation	Degree
1	Prelingual	Severe	35delG/V37I	—–	—–
10	Prelingual	Severe	35delG/35delG	—–	—–
15	Prelingual	Severe	35delG/35delG	—–	—–
23	Prelingual	Severe	35delG/Normal	—–	—–
24	Prelingual	Severe	35delG/35delG	—–	—–
25	Prelingual	Profundo	35delG/35delG	—–	—–
26	Prelingual	Moderate	35delG/35delG	—–	—–
34	Prelingual	Severe	35delG/Delta(GJB6-D13S1830)	brother CI35 35delG/Delta(GJB6-D13S1830)	Severe
39	Prelingual	Moderate	35delG/Normal	—–	—–

IC - Index cases; HL - Hearing loss

## DISCUSSION

In the present study, we found a 27.3% prevalence of the 35delG mutation in the index cases analyzed, 21.2% of alleles with the mutation, and 12.5% in the affected relatives. A prevalence of 3% was also assessed for each one of the mutations V37I and Delta (GJB6-D13S1830), found in the study. These results are concordant with previous studies reported in the literature, conducted in several populations^22^, ^23^, ^24^, ^25^, ^26^. The relative contribution of the 35delG mutation to the non-syndromic hearing loss in these populations varied from 0% (Oman, Korea, Japan) to 70% (Italy, Spain, Greece), demonstrating the genetic heterogeneity among the different countries, even though some of thesestudies were based on a small number of patients, besides the differences in the impairment investigation criteria and the mutation screening methods^23^,^27^, ^28^, ^29^, ^30^.

Recent studies found a 342 thousand base-pair (342 Kb) deletion close to the GJB6 [D(GJB6 - D13S1830)] gene, suggesting that this mutation could cause non-syndromic recessive hearing loss, either by a homozygous deletion or by digenic penetrance of the deletion in the GJB6 gene, associated with a trans mutation in the GJB2 gene in the heterozygous cases^24^. Most genetic cases of hearing impairment result from mutations in a single gene, but an increasing number of cases with two involved genes have been identified31. A multicenter study conducted in nine countries demonstrated that the Delta (GJB6-D13S1830) mutation is more frequent in France, Spain, Israel, the United Kingdom and Brazil, varying from 5.9% to 9.7% of all studied alleles of patients with DFNB1, and being present in about 50% of heterozygous patients in Spain^32^. In the present study, a 3% prevalence of the deletion in the GJB6 gene in heterozygosis with the 35delG mutation was found, which is in agreement with data from the literature^24^,^32^.

In Brazil, a study conducted in newborns from the region of Sao Jose do Rio Preto, SP^33^, found a 2.24% (1:44.6) prevalence of heterozygotes for the 35delG mutation, and another newborn screening, performed in the region of Campinas, SP^34^, found a 0.97% (1:103) prevalence of heterozygotes. In another study, performed in patients with hearing loss, mutations in the GJB2 gene were found in 33.5% of cases, and only the 35delG mutation was identified in 84.2% of mutant alleles^14^. The methodology used in the current study, AS-PCR and automated sequencing, was similar to the three above mentioned researches, but a variation was found in the frequency of the alleles with the 35delG mutation. This finding can be explained by differences in the sample or maybe by the highly heterogeneous ethnic composition of the Brazilian population, with miscegenation among various ethnic groups, mainly Caucasoid and African, allowing the occurrence of differences in prevalence among different regions of the country^35^.

According to the literature, analyses of the GJB2 gene in patients with hearing impairment frequently demonstrate heterozygosis in about 10% to 42% of cases, in spite of the fact that most of the mutations are recessive^23^,^36^,^37^. In this study, we found a 12.1% frequency of heterozygous index cases, a result that agrees with the literature^23^,^36^,^37^.

According to the audiometric results, in the present study the hearing loss was profound in 41.5% of patients, severe in 39.0%, and moderate in 19.5%, with a predominance of the high frequencies (4000-8000 Hz). In patients who were homozygous or heterozygous for 35delG, the moderate-severe degrees of hearing loss were predominant, a pattern that is in agreement with the literature^17^,^38^,^39^. Patients who are homozygous for 35delG display a large variability in the degrees of hearing loss. Most of the autosomal recessive impairments are phenotypically rather consistent, even among sibs, a feature that is not observed for the GJB2-35del gene, mainly in heterozygous cases. This suggests the possibility of other factors modulating the expression of the mutant gene40. An intriguing possibility is that there may be a second conexin gene sharing functions with the GJB2 gene. It is conceivable that a second conexin protein might act as a substitute under certain conditions. Maybe there are modifier genes at other locations or environmental influences which activate or inactivate the promoter regions of the gene. As protein Cx26 is involved in the ion homeostasis of the inner ear, some of these patients may be capable of hearing, for they present a moderate hearing loss, suggesting the existence of alternative or compensatory homeostatic mechanisms. Alterations in protein Cx26 can adversely affect the development of the hearing system, resulting in variations of the degree of hearing loss or assymmetry^38^. Environmental influences, such as noises and ototoxic drugs, can be additive or synergists to the defects caused by mutations in the GJB2 gene, thus increasing the hearing loss^17^,^38^,^41^.

The clinical evaluations of patients in this study do not suggest that environmental causes are the main factors, considering that an autosomal recessive transmission pattern was identified in cases with the 35delG mutation. Moreover, upon audiometric testing, these nine patients presented non-progressive audiometric findings, similar to those of patients described in the literature^23^,^42^, ^43^, ^44^ diagnosed with hearing impairment caused by mutation in the GJB2 gene. This confirms that the gene is involved in the etiology of the hearing loss of patients of this study with a phenotypic expression similar to that described in the literature^23^,^42^, ^43^, ^44^. In approximately one third of the cases of hearing impairment due to mutations in the GJB2 gene, an audiometric pattern of progressive hearing loss is found, as opposed to the two thirds of cases with the typical non-progressive pattern. This means that a child with moderate hearing loss may progress to profound, and the therapies for these two kinds of degrees of hearing loss may be different. Families with a child with moderate-severe or profound hearing loss can be benefited by an analysis of the GJB2^38^ gene.

The 35delG mutation is easy to detect and the test is viable. However, since a great part of the patients with the 35delG mutation are homozygous, broader analyses of the GJB2 gene will be necessary in a high proportion of cases, in order to distinguish the common heterozygous carriers (healthy carriers) from the heterozygous patients with DFNB1. These broader analyses and investigations [including analyses of the entire coding region, the promoter region, the non-coding region of the gene and analyses of the Delta (GJB6-D13S1830) mutation], as performed in the present study, should be oriented by the clinical characteristics of DFNB1^43^,^45^.

According to the literature, molecular tests associated to audiometric data can predict that a significant number of patients with the 35delG mutation will present a moderate-severe hearing loss and others are expected to present profound hearing loss^29^,^44^,^46^, as found in this study. Thus, in spite of the small casuistic, the audiometric pattern was concordant with the literature, enabling us to establish a genotype-phenotype correlation in the ten patients of the sample (9 index cases and 1 affected relative), i.e., the patients with the 35delG mutation presented a moderate-severe to profound, non-progressive hearing loss. A multicenter study is however necessary to verify the true phenotypic expression related to the 35delG mutation in the Brazilian population. Knowledge of the genotype will enable physicians, speech therapists, educators, helped by clinical geneticists, to provide more adequate counseling to parents by evaluating the risk of a future child having a similar hearing impairment. Children diagnosed before six months of age and submitted to a successful treatment with amplification will have a much greater chance of normally developing speech and language.
